# Association of Gestational Hypertension with Anemia under 5 Years Old: Two Large Longitudinal Chinese Birth Cohorts

**DOI:** 10.3390/nu14081621

**Published:** 2022-04-13

**Authors:** Hang An, Huiting Chen, Zhiwen Li, Le Zhang, Yali Zhang, Jianmeng Liu, Rongwei Ye, Nan Li

**Affiliations:** 1 Institute of Reproductive and Child Health/Ministry of Health Key Laboratory of Reproductive Health, Peking University Health Science Center, Beijing 100191, China; anhang@bjmu.edu.cn (H.A.); htchen1999@163.com (H.C.); lizw@bjmu.edu.cn (Z.L.); zhangle@bjmu.edu.cn (L.Z.); zhangyl@bjmu.edu.cn (Y.Z.); liujm@pku.edu.cn (J.L.); 2 Department of Epidemiology and Biostatistics, School of Public Health, Peking University Health Science Center, Beijing 100191, China

**Keywords:** gestational hypertension, anemia, birth cohort, children, infant

## Abstract

Gestational hypertension may interfere with the placental iron metabolism, thus probably increasing the risk of childhood anemia. We aim to examine the association between gestational hypertension and childhood anemia at different ages in two large Chinese birth cohorts. Cohort 1 was conducted in 5 counties in northern China and was comprised of 17,264 mother–children pairs (97.3%) during 2006–2009, whereas cohort 2 was conducted in 21 counties in southern China and was comprised of 185,093 mother–children pairs (93.8%) during 1993–1996. All pregnant women were registered in a monitoring system and followed up until the termination of pregnancies. The childhood anemia was diagnosed at 6 month and 12 month in cohort 1 and at 55 month in cohort 2. The overall incidence of childhood anemia was 6.78% and 5.28% at 6 month and 12 month, respectively, in cohort 1 and 13.18% at 55 month in cohort 2. Gestational hypertension was associated with increased risk of anemia at 6 month (adjusted Odds Ratio (OR): 1.31; 95% confidence interval (CI): 1.05, 1.63) and at 12 month (adjusted OR: 1.50; 95% CI: 1.18, 1.90) in cohort 1 and at 55 month (adjusted OR: 1.06; 95% CI: 1.01, 1.12) in cohort 2. The hemoglobin values of children at different ages were lower among gestational hypertension group in the linear models, which was consistent with the results of binary regression analysis. Our study found gestational hypertension may associate with an increased risk of childhood anemia. It suggests a possible need for exploring changes in prenatal care that might prevent childhood anemia.

## 1. Introduction

Anemia is one of the most common nutritional disorders among preschool children worldwide, particularly in developing countries. The estimated prevalence of anemia under 5 years old was 47.4% globally, which affected 293 million children [1]. Anemia during childhood may lead to an increased risk of morbidity and mortality in younger age [2]. It is also associated with impaired neurological development and may cause cognitive damage in the long run [3,4]. Several factors may relate to childhood anemia such as genetic and malnutrition status of mother and child [5]. However, the potential etiology of anemia still remains unclear.

It is estimated that iron deficiency is the leading cause of childhood anemia [6]. Impaired placental function may cause limited iron store and transfer, hence increasing the risk of anemia [7,8,9]. Gestational hypertension, defined as de novo hypertension, complicates 5–10% of all pregnancies [10]. Previous studies indicated women with hypertensive disorders of pregnancy might have placental structural changes which have effects on maternal–fetal transport of nutrients, including iron [11,12]. Both ferritin and calculated iron storage values of infants, markers used to evaluate the iron storage status, were less in the gestational hypertension group compared with the normal group. Yet, some studies indicated that infants might not be affected by anemia, though the iron storage was abnormal [13]. As former studies had conflicting results and suffered from small sample size to detect the effects, we need more evidence to specifically examine the relationship between gestational hypertension and childhood anemia.

During the first 4–6 months of postnatal life, the endogenous iron stores of healthy infants will cover their demands for growth [14]. For infants with mothers who have gestational hypertension, the iron storage condition may be affected and these influences may exist hereafter. However, it still remains unclear whether the association between maternal hypertension during pregnancy and anemia exists for children. In recent years, the theory of “developmental origins of health and disease” (DOHaD) underlies the role of maternal health status on trajectory health development [15]. Although China has made a series of nutrition improvement projects for children over the past 30 years, maternal factors should be paid more attention. Therefore, we used two large Chinese birth cohort studies to investigate the association of gestational hypertension with childhood anemia at different times.

## 2. Methods

### 2.1. Background and Subjects for Current Study

Cohort 1 originated from a randomized controlled trial aiming to evaluate the effect of micronutrient supplementation during pregnancy on pregnant women and fetuses. This study was implemented in 5 rural counties in Hebei province of northern China. Briefly, 18,775 primiparous women were enrolled before 20 weeks of gestation from 2006 to 2009 and were randomly assigned to 3 groups (folic acid, folic acid plus iron, and multiple micronutrients) [16]. Of 18,775 women, all of them had hemoglobin concentrations ≥ 100 g/L and 17,748 women had live singleton births with available data registered on the monitoring system of both prenatal information and offspring examination records. We further excluded 43 (0.24%) women with multi-fetal gestations; 450 (2.54%) infants had no hemoglobin records during 5–7 month or 11–13 month. After these exclusions, 17,264 women (97.3% of the targeted population) from cohort 1 were finally included in the analysis.

**Figure 1:**
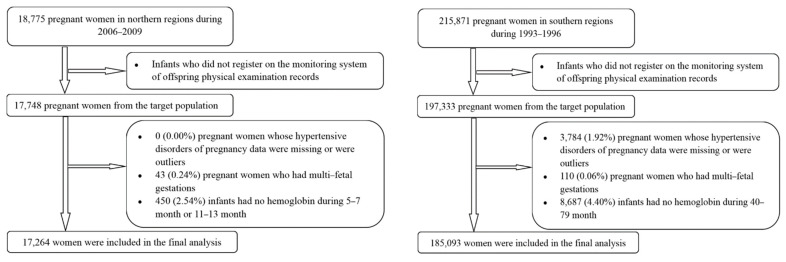
Flowchart of participants in cohort 1 (**left**) and cohort 2 (**right**).

Cohort 2 was based on a large prospective cohort study aiming to study the effect of preconceptional use of folic acid on neural tube defects, as well as child growth and development. It was implemented in 21 cities or counties in Zhejiang and Jiangsu provinces of southern China. Briefly, of 215,871 women who prepared for marriage or became pregnant registered in a perinatal health care surveillance system from 1993 to 1996 [17]. Further, 197,333 of their children were followed up in 2000 to obtain the information of physical examinations. Of these women, we excluded 3784 (1.92%) for whose gestational hypertensive disorders of pregnancy records were unknown outliers (systolic blood pressure value < 60 mm Hg or >200 mm Hg; diastolic blood pressure value < 40 mm Hg or >164 mm Hg); 110 (0.06%) with multi-fetal gestations and 8687 (4.40%) whose infants did not have hemoglobin records during 40–79 month. After these exclusions, 185,093 women (93.8% of the targeted population) from cohort 2 were finally included in the analysis (Figure 1).

The original study was approved by the Peking University Health Science Center Institutional Review Board. The secondary analyses of already collected data were deemed exempt by the institutional review board.

### 2.2. Exposure and Covariates

We collected data through the same perinatal health care surveillance systems in both birth cohorts. At each visit, blood pressure in the right arm was measured by trained health workers. They measured with a mercury sphygmomanometer and recorded on two or more consecutive occasions with an interval of ≥6 h. We defined gestational hypertension as an absolute blood ≥ 140/90 mm Hg after 20 weeks of gestation, or as a blood pressure increment of ≥30/15 mmHg after 20 weeks of gestation as compared with the first trimester [18]

In addition to gestational hypertension, other covariates were collected with mainly consistent structured questionnaire. The information was first filled out in a brochure by health workers, then entered into computers by trained staff in hospitals and finally transferred to the Peking University project center. The covariates drawn from surveillance systems included continuous covariates: maternal age (years), body mass index (BMI, kg/m^2^) at first prenatal visit, anemia during pregnancy (maternal hemoglobin concentration < 110 g/L at any time during pregnancy); and age at offspring hemoglobin measurement (months); categorical covariates: education (high school or higher, junior high school, primary school or lower, or unknown), occupation (farmer or other), ethnicity (Han or other), after 42 days postpartum feeding practices (exclusive breastfeeding or others), anemia during pregnancy (yes or no), micronutrient supplementation (folic acid, iron–folic acid, and multiple micronutrients) for cohort 1, parity (primigravida or multigravida) and periconceptional folic acid consumption status (yes or no) for cohort 2.

### 2.3. Hemoglobin Measurement

Before study initiation, all doctors were trained to measure hemoglobin according to standard procedures. A step-by-step instruction leaflet was pasted on the wall of doctors’ rooms to ensure the compliance. To minimize testing bias, the doctors’ rooms were equipped with heating devices in winter to maintain temperature at over 18 °C. In cohort 1, infants’ hemoglobin was measured by using capillary blood via the HemoCue system (HemoCue AB, Angelholm, Sweden). In cohort 2, children’s hemoglobin was measured with a standard cyanmethemoglobin method by using capillary blood via devices available at each hospital; two commonly used devices were the visible spectrophotometer and hemoglobinometer (model 721, China). All project hospitals were provided with standard hemoglobin solutions (50, 100, 150 and 200 g/L) and with a step-by-step procedure for calibrating the hemoglobinometer and for preparing the standard curve for the 721 visible spectrophotometer. We defined anemia as hemoglobin concentrations < 110 g/L for infants and children aged < 60 month, and concentrations < 150 g/L for children aged ≥ 60 month, based on WHO recommendations [19].

### 2.4. Statistical Analysis

We compared the characteristics of women between gestational hypertension and non-gestational hypertension subjects in the mean age and BMI, ethnicity, education, occupation, anemia during pregnancy, feeding practices and age at follow-up visit. We used the Student’s *t*-test for quantitative variables and the χ^2^ test for categorical variables. Logistic regression models were used to estimate crude and adjusted odds ratios (ORs) after adjusting for the main underlying confounders such as maternal age, BMI, education, occupation, ethnicity, anemia during pregnancy and week of gestation at hemoglobin measurement. One additional confounder for cohort 1 was micronutrient supplementation; additional confounders for cohort 2 were parity and folic acid intake. We further used the linear regression model to estimate the crude and adjusted mean difference in hemoglobin for different groups. Modification of the effect of gestational hypertension by covariates was examined by adding an interaction term to the multivariable logistic regression model. The effect of gestational hypertension was subsequently estimated by multilogistic regression in strata of maternal characteristics with *p*-values < 0.1 for interaction. All data were analyzed using SPSS for Windows software (ver. 20.0; SPSS Inc, Chicago, IL, USA).

## 3. Results

Of the 17,264 women included in cohort 1, 1105 (6.40%) had gestational hypertension, whereas of the 185,093 women in cohort 2, 17,677 (9.55%) had gestational hypertension. Table 1 shows maternal and children characteristics according to gestational hypertension. For both cohorts, women with gestational hypertension were more likely to be older, have a non-farmer occupation, have higher BMI and had a lower proportion of exclusive breastfeeding. Most women were educated to a junior high or higher school level in both cohorts. The percentage of women with anemia during pregnancy in gestational hypertension group was similar with non-gestational hypertension group in cohort 1, whereas the percentage was higher in gestational hypertension group in cohort 2. Mean age at follow-up visit for children with mother of gestational hypertension was younger than those without gestational hypertension in cohort 1, whereas the age of children was similar across two groups in cohort 2.

The total incidences of anemia at 6 month (1171) and 12 month (911) in cohort 1 were 6.78% and 5.28%, respectively, and 13.18% at 55 month (24,403) in cohort 2. The associations between gestational hypertension and children’s anemia at different ages is demonstrated in Table 2. In both cohorts, the incidence of childhood anemia was higher in women with gestational hypertension. All crude analysis revealed gestational hypertension was associated with an increased risk of childhood anemia. After adjustment for confounders, associations of gestational hypertension with anemia at 6, 12, and 55 month remained almost unchanged (ORs (95% CI): 1.31 (1.05, 1.63), 1.50 (1.18, 1.90), and 1.06 (1.01, 1.12), respectively).

We further compared the mean hemoglobin concentration of children according to their mother’s gestational hypertension status in Table 3. Mean (±SD) hemoglobin concentration was 121.71 ± 8.67 g/L and 122.07 ± 8.18 g/L at 6 month and 12 month, respectively, in cohort 1, and was 119.52 ± 10.19 g/L at 55 month in cohort 2. Gestational hypertension was associated with significant reduction of hemoglobin concentrations in both cohorts, which was consistent with results of childhood anemia. The adjusted change of hemoglobin at 6, 12 and 55 month were −1.12 g/L (95% CI: −1.65, −0.59 g/L), −1.48 g/L (95% CI: −1.98, −1.00 g/L) and −0.20 g/L (95% CI: −0.37, −0.03 g/L) among children born to mothers with gestational hypertension relative to those without.

In the analysis of effect modification, occupation as a maternal characteristic was a significant interaction term. When the analysis was stratified by occupation, we observed gestational hypertension might increase the risk of anemia for women with farmer occupation, but not for non-farmer occupation in both cohorts (Table 4).

## 4. Discussion

In these two large longitudinal Chinese birth cohorts, we found gestational hypertension was positively associated with childhood anemia at 6 month, 12 month and 55 month. Children’s hemoglobin in the gestational hypertension group was significantly less than the normal group across different ages. These findings help to acquire a better understanding about the effect of hypertension management during pregnancy on prevention of childhood anemia.

Several studies have investigated the association between hypertensive disorders of pregnancy and the occurrence of infant anemia [11,12,13,20,21]. Some studies demonstrated that infants born to mothers with maternal hypertension had lower ferritin levels and iron stores, which reflected offspring’s potential risk of having anemia [11,12,13]. One Korean study [12] compared the iron status of newborn infants and found serum ferritin of appropriate gestational age infants from mother with hypertensive disorders of pregnancy group significantly lower than normal group (median (interquartile range, IQR): 108.5 (46.8–184.8) ng/mL versus 143.0 (88.0–235.0) ng/mL). Similar results were found in the comparison of total body iron stores of the infants. However, this study used retrospective data and only enrolled specific neonatal individuals which may lead to selection and recall bias. Another cohort study [13] also found ferritin values in children aged 0.5 to 1 year were inversely associated with adverse maternal factors after adjustment for some covariates (*β*: −0.330, *p*-value: 0.01). However, neither iron deficiency (ferritin < 12 mg/μg) nor iron deficiency anemia (iron deficiency and hemoglobin < 110 g/L) were different according to the presence or absence of hypertension groups. Negative association also found in other studies [20,21]. Yet, these studies were all conducted in developed countries and children had better nutrition status. Meanwhile, these studies had a small sample size (number of anemia < 50) which might not have the capacity to detect the effects.

Our study combined two large birth cohorts to compare the effect of gestational hypertension on childhood anemia at different times. In cohort 1, we measured the children’s hemoglobin twice at 6 month and 12 month, respectively. We found the effect of gestational hypertension existed consistently in children aged 6 month, 12 month and even 55 month. Uijterschout et al. [13] used data in one time examination but across different ages and indicated the difference of children’s ferritin levels across hypertension and normal groups was not observed after the age of 12 month. Conflicting results may be due to the heterogeneity of population and study design. The effect of gestational hypertension significantly decreased at 55 month (adjusted OR = 1.06 (95% CI: 1.01, 1.12)). One possible explanation was that children might absorb nutrition through more pathways such as formula and cow’s milk. The richer dietary pattern might attenuate the adverse effect of gestational hypertension [22]. Interestingly, we noted the risk ratio of hypertension was not decreased at 12 month compared with 6 month. Although the number of children with childhood anemia decreased, the number of anemic children with non-gestational hypertension mother decreased more greatly. This suggested gestational hypertension might have a greater effect at early age despite the supplement of nutrition. The health condition of these children should be paid more attention. Further studies were needed to provide more evidence.

Few studies investigated the interaction effects between gestational hypertension and maternal characteristics. Previous studies revealed that mother’s occupation may have an effect on childhood anemia [23,24]. A study conducted in Ghana [24] showed children whose mothers were farmers were less likely to have anemia (adjusted OR = 0.17 (95% CI: 0.05–0.60)). However, the possible joint effect of occupation was less discussed. Our findings indicated that the mother’s occupation modified the association between gestational hypertension and childhood anemia. The negative associations were stronger in the farmer group across different times.

The exact mechanisms of the association between gestational hypertension and childhood anemia still remain unknown. However, the existing evidence provides the possibility that placental dysfunction might be involved. Several recent studies showed that higher blood pressure during pregnancy was associated with modifications of placenta DNA methylation [25,26], hence, it might have an effect on placental functions. The placenta plays an important role in storing and transporting iron and nutrients [27,28]. Maternal gestational hypertension might relate to impaired placental function and decreased placental perfusion [29,30]. These conditions restricted the amount of available iron to the fetus and may result in infant anemia.

This study had several strengths. First, we used two large birth cohorts to investigate the association. The population in these cohorts represented different regions of China, cohort 1 for northern China and cohort 2 for southern China. They had different living conditions and various maternal characteristics; however, we reached the same conclusions, which made our results more reliable. Meanwhile, we conducted the uniformly procedure to collect and manage data. The blood pressure and hemoglobin were all measured by trained health workers and diagnosed by specialists, which minimized the risk of misclassification bias. The use of a surveillance system guaranteed that over 90% of the mother–child information could be followed, which subsequently enabled us to detect the effects with a large sample size. Furthermore, our study conducted repeated measurements of hemoglobin at different times, which provided the chance to explore the long-term effect of gestational hypertension.

Some limitations should be considered when the results are interpreted. Limited by local health disparities, we used the different devices to measure children’s hemoglobin values in these two cohorts. As the difference of measurement methods between these two devices was not evaluated, it may lead to the misclassification of anemic outcomes. Some potential confounding information, such as maternal smoking and alcohol drinking, were not collected. However, smoking and alcohol use were both rare among women in rural China, especially among pregnant women at the time of our study. We did not measure the iron status and iron indicator such as serum ferritin and transferrin in this study; therefore, we could not conclude the effects of different types of anemia on childhood anemia. Further studies are needed to explore the possible mechanisms that iron plays a part in. The gestational age of when anemia during pregnancy and gestational hypertension were diagnosed was unavailable in this study. The diagnosis gestational age might affect the medical intervention afterwards, and hence influence the association. Additionally, the participants in our study were Han (China’s predominant ethnic group), so our results may not be generalizable to other populations.

## 5. Conclusions

In conclusion, we used two large birth cohort studies in China to investigate the association of gestational hypertension and childhood anemia. After adjusting for potential confounders, we found that gestational hypertension could increase the risk of anemia at 6 month, 12 month and 55 month. Considering the possible long-term effect brought by gestational hypertension, the management of blood pressure during pregnancy should be paid more attention to prevent the anemia under 5 years old.

## Figures and Tables

**Table 1:** Maternal and child characteristics according to gestational hypertension group.

Characteristics	Cohort 1				*p*	Cohort 2				*p*
	Gestational Hypertension Group (*n* = 1105)		Non-Gestational Hypertension Group (*n* = 16,159)			Gestational Hypertension Group (*n* = 17,677)		Non-Gestational Hypertension Group (*n* = 167,416)		
	*n*	%	*n*	%		*n*	%	*n*	%	
**Mother**										
Age (years, mean ± SD)	23.74 ± 3.14		23.36 ± 2.80		<0.001	25.01 ± 3.36		24.84 ± 3.19		<0.001
Body mass index (kg/m^2^, mean ± SD)	23.39 ± 3.68		22.21 ± 2.77		<0.001	20.82 ± 2.43		20.51 ± 2.26		<0.001
Han ethnic group	1095	99.10	15,964	98.79	0.472	17,532	99.18	165,994	99.15	0.726
Education					0.001					0.095
High school or higher	246	22.26	2873	17.78		1765	9.98	17,542	10.48	
Junior high school	840	76.02	13,028	80.62		10,492	59.35	99257	59.29	
Primary school or lower, or unknown	19	1.72	258	1.60		5420	30.66	50,617	30.23	
Farmer occupation	969	87.69	14,737	91.20	<0.001	10,230	57.87	100,509	60.04	<0.001
Exclusive breastfeeding	1039	94.03	15,460	95.67	0.011	15,097	85.40	146,063	87.25	<0.001
Anemia during pregnancy	83	7.51	997	6.17	0.202	11,684	66.10	107,095	63.97	<0.001
**Child**										
Hemoglobin concentration	120.62 ± 8.74 *		121.79 ± 8.66 *		<0.001 *	119.25 ± 10.26		119.54 ± 10.19		<0.001
(g/L, mean ± SD)	120.66 ± 8.11 ^†^		122.17 ± 8.17 ^†^		<0.001 ^†^					
Age at follow-up visit (months, mean ± SD)	6.23 ± 0.47 *;		6.26 ± 0.44 *;		0.009 *	55.61 ± 8.41		55.50 ± 8.19		0.096
	12.23 ± 0.46 ^†^		12.27 ± 0.43 ^†^		0.020 ^†^					

SD, standard deviation. * First follow-up visit. ^†^ Second follow-up visit.

**Table 2:** Crude and adjusted RRs of anemia for gestational hypertension group compared with non-gestational group.

	Children with Anemia							
	Gestational Hypertension Group		Non-Gestational Hypertension Group					
Mean Age at Follow-Up	*n*	%	*n*	%	Crude OR (95% CI)	*p*	Adjusted OR (95% CI) *	*p*
**Cohort 1**								
6 month	98	8.87	1073	6.64	1.37 (1.10, 1.70)	0.005	1.31 (1.05, 1.63)	0.016
12 month	83	7.51	828	5.12	1.50 (1.19, 1.90)	0.001	1.50 (1.18, 1.90)	0.001
**Cohort 2**								
55 month	2477	14.01	21,926	13.10	1.08 (1.03, 1.13)	0.001	1.06 (1.01, 1.12)	0.016

OR, odds ratio; CI, confidence interval. * Common confounders adjusted for in the multiple logistic regression for both cohorts included maternal age, BMI, education, occupation, ethnicity, feeding practices, anemia during pregnancy and week of gestation at hemoglobin measurement. One additional confounder for cohort 1 was micronutrient supplementation; additional confounders for cohort 2 were parity and folic acid intake.

**Table 3:** Crude and adjusted mean difference in hemoglobin (g/L) for gestational hypertension group compared with non-gestational group.

	Gestational Hypertension Group	Non-Gestational Hypertension Group				
Mean Age at Follow-Up	Mean ± SD (g/L)	Mean ± SD (g/L)	Crude Mean Difference (95% CI)	*p*	Adjusted Mean Difference (95% CI) *	*p*
**Cohort 1**						
6 month	120.62 ± 8.74	121.79 ± 8.66	−1.17 (−1.70, −0.64)	<0.001	−1.12 (−1.65, −0.59)	<0.001
12 month	120.66 ± 8.11	122.17 ± 8.17	−1.50 (−2.00, −1.00)	<0.001	−1.48 (−1.98, −1.00)	<0.001
**Cohort 2**						
55 month	119.25 ± 10.26	119.54 ± 10.19	−0.29 (−0.45, −0.13)	<0.001	−0.20 (−0.37, −0.03)	0.022

CI, confidence interval. * Common confounders adjusted for in the multiple logistic regression for both cohorts included maternal age, BMI, education, occupation, ethnicity, feeding practices, anemia during pregnancy and week of gestation at hemoglobin measurement. One additional confounder for cohort 1 was micronutrient supplementation; additional confounders for cohort 2 were parity and folic acid intake.

**Table 4:** Stratified analysis of the effects of gestational hypertension on children with anemia by occupation.

	Children with Anemia						
	Gestational Hypertension Group		Non-Gestational Hypertension Group				
Maternal Characteristics	*n*	%	*n*	%	Crude OR (95% CI)	Adjusted OR (95% CI) *	*p* for Interaction
Cohort 1—6 month							0.010
Farmer occupation	87	8.98	963	6.53	1.41 (1.12, 1.78)	1.32 (1.05, 1.67)	
Non-farmer occupation	11	8.09	110	7.74	1.05 (0.55, 2.00)	1.21 (0.63, 2.34)	
Cohort 1—12 month							0.001
Farmer occupation	76	7.84	756	5.13	1.57 (1.23, 2.01)	1.55 (1.21, 1.98)	
Non-farmer occupation	7	5.15	72	5.06	1.02 (0.46, 2.26)	1.01 (0.45, 2.28)	
Cohort 2							<0.001
Farmer occupation	1530	14.96	13,575	13.51	1.13 (1.06, 1.19)	1.10 (1.03, 1.17)	
Non-farmer occupation	947	12.72	8351	12.48	1.02 (0.95, 1.10)	1.01 (0.93, 1.09)	

OR, odds ratio; CI, confidence interval. * Common confounders adjusted for in the multiple logistic regression for both cohorts included maternal age, BMI, education, ethnicity, feeding practices, anemia during pregnancy and week of gestation at hemoglobin measurement. One additional confounder for cohort 1 was micronutrient supplementation; additional confounders for cohort 2 were parity and folic acid intake.

## Data Availability

The data are available in the main text, or can be obtained by contacting the corresponding author (Nan Li).
